# Competitive Biosynthesis of Bacterial Alginate Using *Azotobacter vinelandii* 12 for Tissue Engineering Applications

**DOI:** 10.3390/polym14010131

**Published:** 2021-12-30

**Authors:** Andrei A. Dudun, Elizaveta A. Akoulina, Vsevolod A. Zhuikov, Tatiana K. Makhina, Vera V. Voinova, Nikita V. Belishev, Dolgor D. Khaydapova, Konstantin V. Shaitan, Garina A. Bonartseva, Anton P. Bonartsev

**Affiliations:** 1 Research Center of Biotechnology of the Russian Academy of Sciences Leninsky Ave, 33, Bld. 2, 119071 Moscow, Russia; dudunandrey@mail.ru (A.A.D.); vsevolod1905@yandex.ru (V.A.Z.); tat.makhina@gmail.com (T.K.M.); bonar@inbi.ras.ru (G.A.B.); 2 Faculty of Biology, M.V. Lomonosov Moscow State University, Leninskie Gory 1-12, 119234 Moscow, Russia; akoulinaliza@gmail.com (E.A.A.); veravoinova@mail.ru (V.V.V.); nbelishev@gmail.com (N.V.B.); shaytan49@yandex.ru (K.V.S.); 3 Department of Soil Physics and Reclamation, Soil Science Faculty, M.V. Lomonosov Moscow State University, Leninskie Gory 1-12, 119234 Moscow, Russia; dkhaydapova@yandex.ru

**Keywords:** bacterial alginate, competitive biosynthesis, *Azotobacter vinelandii*, full factorial design, poly(3-hydroxybutyrate), cytotoxicity, tissue engineering

## Abstract

This study investigated the effect of various cultivation conditions (sucrose/phosphate concentrations, aeration level) on alginate biosynthesis using the bacterial producing strain *Azotobacter vinelandii* 12 by the full factorial design (FFD) method and physicochemical properties (e.g., rheological properties) of the produced bacterial alginate. We demonstrated experimentally the applicability of bacterial alginate for tissue engineering (the cytotoxicity testing using mesenchymal stem cells (MSCs)). The isolated synthesis of high molecular weight (M*_w_*) capsular alginate with a high level of acetylation (25%) was achieved by FFD method under a low sucrose concentration, an increased phosphate concentration, and a high aeration level. Testing the viscoelastic properties and cytotoxicity showed that bacterial alginate with a maximal M*_w_* (574 kDa) formed the densest hydrogels (which demonstrated relatively low cytotoxicity for MSCs in contrast to bacterial alginate with low M*_w_*). The obtained data have shown promising prospects in controlled biosynthesis of bacterial alginate with different physicochemical characteristics for various biomedical applications including tissue engineering.

## 1. Introduction

To date, the use of bacterial alginates and polyhydroxyalkanoates (PHAs) has attracted researchers’ attention whose main research focuses are tissue engineering and biopharmacology [1,2]. PHAs are semi-crystalline hydrophobic polyesters, where the crystalline phase dominates over the amorphous [3]. Alginates exist as hydrophilic unbranched exopolysaccharides with two uronic acid monomers: (1-4) -β-d-Mannuronic acid (M) and its C5 epimer α-l-Guluronic acid (G) [4]. These polymers are biocompatible and biodegradable and can be used to successfully develop 3D constructs for tissue engineering and regenerative surgeries [5]. Since biosynthesis and bacterial PHA physicochemical properties studies were discussed in detail, this study will be mainly focused on bacterial alginates [3,6]. The distinctive ability of this polymer is the ionotropic interaction of guluronic residues with bivalent calcium cations leading to hydrogel formation [7]. These polymers have great potential for developing numerous materials such as hydrogels and scaffolds in tissue engineering and regenerative medicine [8,9], and wound dressings with biologically active encapsulated compounds [2] (e.g., peptides, proteins [5], vector genetic constructs and etc.) [10].

Some bacterial species of the *Azotobacter* sp. genus are capable of simultaneously synthesizing alginate and PHAs. These microorganisms can synthesize poly(3-hydroxybutyrate) (PHB) to almost 80% of a bacterial cell dry weight. Also, these bacteria, in addition to PHB production, can also synthesize alginates [11]. Unlike brown algae (which usually serve as an alginate source in the industry), the *Azotobacter* sp. genus bacteria have several advantages to its synthesis [12]. Firstly, some of the bacterial alginate physicochemical properties differ from algal alginate (e.g., O-acetylation in bacterial alginates at C_2_ and C_3_ positions in mannuronic acids) [13]. O-acetylation affects viscosity, calcium ion interactions and a reaction of mannuronan epimerase, and mannuronan lyase [14,15]. Thus, acetyl groups can directly affect the polymer’s physicochemical properties. Secondly, bacterial cultivation in laboratory conditions makes it possible to control and regulate the alginate synthesis obtaining the polymer in a variety of physicochemical properties [9,11]. In contrast, commercial algal alginates polymer synthesis has limited regulation freedom, since the monomeric composition and M*_w_* of alginates depend on the conditions at which large marine algae grow. When algae grow in natural conditions, it becomes more difficult to regulate factors such as temperature, light intensity, etc. Also, it is difficult to culture it in artificial conditions [16,17]. Thirdly, the alginate biosynthesis using the *Azotobacter* genus bacteria is preferable to the *Pseudomonas* genus since the latter are pathogenic microorganisms [18]. All of these aspects make *Azotobacter* sp. almost an ideal candidate for synthesis of the bacterial alginate, which can be further used as a stabilizer, thickener, and a gelling or film-forming agent for various applications in the food and medical industries [19,20].

**Figure 1:**
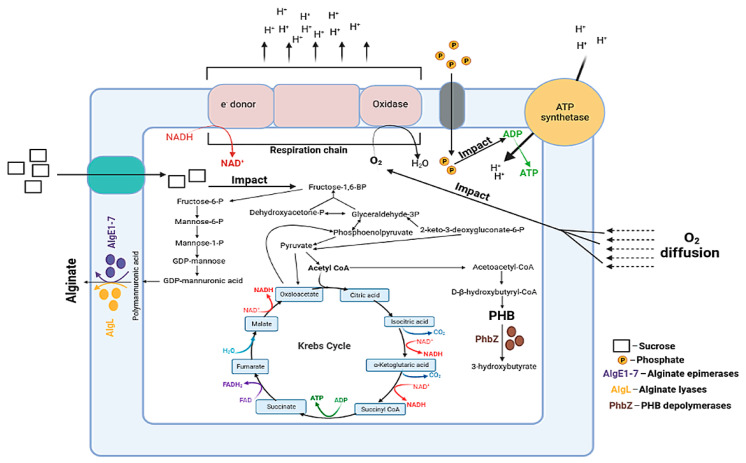
Metabolic pathways of the alginate and poly(3-hydroxybutyrate) biosynthesis.

Metabolic pathways of the alginate and PHB biosynthesis in bacteria *Azotobacter* sp. are closely intertwined with each other (Figure 1) [19].

The metabolic pathway of alginate synthesis consists of a cascade of enzymatic reactions, starting from dehydrogenase (algD), followed by polymerization of the chain (alg8) [19,21,22,23] and terminating with various modifications of the polymannuron chain (MM) using acetylase complexes and epimerization, wherein mannuron residues get converted into guluronic [24,25,26]. The PHB synthesis consists of three stages: (1) it begins with the condensation of two acetyl-CoA molecules using the β-ketothiolase enzyme; (2) then, acetoacetyl-CoA reductase converts acetoacetyl-CoA into 3-hydroxybutyryl-CoA; (3) finally, PHB chain polymerization with the PHB synthase enzyme occurs [19,27,28].

In our previous investigations, all strains of the *Azotobacter* genus were assessed for the alginate synthesis and, as a result, *A. vinelandii* 12 demonstrated the most evident indications of the alginate production. Therefore this strain was chosen for the experiment [29]. In this study, the aim was to optimize the conditions of the alginate biosynthesis using the *Azotobacter vinelandii* 12 producing strain. In various studies the PHB biosynthesis via the *Azotobacter* sp. genus was observed in detail, where most of the attention was paid to oxygen, phosphates, and the influence of various carbon sources’ concentrations as the main regulatory factors [16,30,31,32]. However, in the case of bacterial alginate, quite a few studies deal with the influence of cultivation factors on its production. Moreover, in studies on the biosynthesis of bacterial alginates, the possibility of their biomedical use is very rarely demonstrated experimentally. On the other hand, to date, the use of alginate from algae as a biomaterial is growing rapidly in the medical industry and biomedical research, as well as tissue engineering [2,33,34]. For instance, our recent study demonstrated that PHB/alginate/hydroxyapatite scaffolds promoted enhanced regeneration of critical-sized bone defects [35]. In addition to bone tissue, alginate-based biomaterials are also widely used for regeneration or drug delivery in the skin, cartilage, osteochondral and cardiac tissue [36,37,38,39]. Thus, the novelty of our investigation is the optimization of the bacterial alginate biosynthesis, including separated synthesis of different types of alginate with various physicochemical properties in a wide range and low cytotoxicity (which is of great value for tissue engineering applications).

The aim of this study was to assess the impact of the following factors: sucrose, phosphates, and aeration on the alginate and PHB biosynthesis using the bacterial strain *Azotobacter vinelandii* 12 via the full factorial design (FFD) method and to demonstrate experimentally the applicability of bacterial alginate for tissue engineering.

## 2. Materials and Methods

### 2.1. Materials

Ethylenediaminetetra-acetic acid (EDTA), Sodium hydroxide (NaOH), components of the culture medium: dipotassium phosphate (K_2_HPO_4_·3H_2_O), magnesium sulfate (MgSO_4_·7H_2_O), sodium chloride (NaCl), sodium molybdate (Na_2_MoO_4_·2H_2_O), calcium carbonate (CaCO_3_), iron (II) sulfate (FeSO_4_·7H_2_O), sodium citrate, calcium chloride (CaCl_2_), monopotassium phosphate (KH_2_PO_4_), sucrose, agar, sodium alginate from brown algae; all reagents were obtained from Merck (former Sigma-Aldrich, Darmstadt, Germany). The set of reagents for sequencing the 16S gene included: kits “Cleanup Standard”, “Extract DNA Blood”, primers fD1 and rD1 (Evrogen, Moscow, Russia). All chemicals used in this study were of analytical grade.

### 2.2. Producer Strain

The object of the study was the *Azotobacter vinelandii* 12 strain isolated from soddy-podzolic soil located in Moscow region (Russia). The strain was maintained in the laboratory of biochemistry of nitrogen fixation and nitrogen metabolism of the Bach Institute of Biochemistry of the Federal Research Center “Fundamental Foundations of Biotechnology” of the Russian Academy of Sciences [11].

### 2.3. 16S Analysis

To confirm that the species’ identity was *A. vinelandii*, the 16S rRNA gene was sequenced. To achieve this, primers fD1—(5′-AGAGTTTGATCCTGGCTCAG-3′) and rD1—(5′-AAGGAGGTGATCCAGCC-3′) were selected using the primer-blast tool (https://www.ncbi.nlm.nih.gov/tools/primer-blast/, accessed on 15 January 2020). To amplify the 16S gene, a standard reagent set for setting up a PCR reaction was used (namely thermostable Tag-polymerase, a mixture of deoxynucleotide triphosphates (dNTPs)) and 10-fold Tag buffer with the addition of Mg^2+^ ions (30 mM) and milli-Q water; all reagents were purchased from Evrogen (Moscow, Russia). After amplification on a T100 Thermal Cycler (Bio-rad, Hercules, CA, USA), the PCR product was purified using the Cleanup Standard kit (Evrogen, Moscow, Russia). The resulting 16S amplicon was sequenced on a 3730xl equipment (Thermofisher Scientific, Waltham, MA, USA). After sequencing, sequence reads were screened for chimeras and assembled using the DNA Baser Assembler 5.15.0 software. For species identification, the assembled contig was compared with the other 16S genes in the NCBI Genbank database (https://blast.ncbi.nlm.nih.gov/Blast.cgi, accessed on 23 January 2020) using the BLASTN tool.

### 2.4. Preparation of Inoculum

In order to maintain the *A. vinelandii* 12 bacteria culture, the following composition of Ashby’s solid nutrient medium was used: 200 mg/L K_2_HPO_4_, 200 mg/L MgSO_4_, 200 mg/L NaCl, 6 mg/L Na_2_MoO_4_, 5.0 g/L CaCO_3_, 20.0 g/L sucrose, 20.0 g/L agar. Inoculum *A. vinelandii* 12 was grown on liquid Burke’s medium in shaker flasks with a volume of 750 mL (200 mL of medium) at 250 rpm; the initial pH of the medium: 7.2; cultivation temperature: 28 °C; seeding duration: one day; the volume of the applied seeding: 4%.

### 2.5. Cultivation Conditions

To assess the influence of cultivation conditions for setting up the FFD method on the alginate biosynthesis and PHB several varieties of Burk’s medium were used. The initial pH of the medium was 7.2; cultivation temperature, 28 °C; the volume of the applied seeding, 4%. Cultivation was carried out in 750 mL shaker flasks (the volume of the culture medium was 200 mL). The optical density of the culture medium was determined nephelometrically at 520 nm. The morphology of the bacterial strain was investigated via light microscopy using a Biomed-1 microscope (Biomed, Moscow, Russia) with a digital camera. Fuchsin staining was used to visualize cells and alginate (Supplemental Materials, Figure S1).

In this study, the sucrose and phosphate (K_2_HPO_4_) concentrations and different agitation rates of the culture (O_2_) in rpm (Innova 43 microbiological rocking chair (New Brunswick Scientific, Enfield, CT, USA)) were used as factors (Table 1).

The FFD method was carried out using three parameters (sucrose, phosphates, and agitation rate (oxygen)), where each parameter was considered as a two-level unit (2^3^): an upper level (+), which corresponds to high values of concentrations and a lower level (**−**), corresponding to low values of concentrations [40]. The use of a two-level factorial analysis is the most relevant method for a clear understanding of the key factors that determine the negative and positive effects on polymer biosynthesis. Each experiment was performed in three replications [40].

### 2.6. Isolation and Purification of Alginate and PHB

Two types of alginate were isolated from *A. vinelandii* 12 biomass: free and capsular (see Section 3.3 in Results). Isolation of free alginate was achieved by separating biomass and cultural medium via centrifugation at 11,000× *g* for 30 min under 4 °C. Then, the supernatant was precipitated with three volumes of ethanol cooled to −20 °C, where the resulting alginate precipitates were collected and dried via lyophilization using Alpha 1-2 LD plus (Martin Christ, Osterode am Harz, Germany). Capsular alginate was isolated from biomass by dissolving it in 1 M NaCl and 100 mM EDTA at a ratio of 1:8:1 (biomass:NaCl:EDTA). Then the sample was incubated for 1 h at 60 °C accompanied by stirring on an orbital shaker PSU-20i (Biosan, Riga, Latvia) for complete homogenization of the solution. Afterward, the supernatant was obtained by centrifugation at 11,000× *g* for 30 min. Subsequently, three volumes of ethanol were added to the supernatant and the precipitate was collected and lyophilized. At the final stage of purification, dry precipitates of free and capsular alginates were dissolved in 1 M NaCl and dialyzed against 1 L of 0.1 M NaCl for 30 h under 4 °C. To obtain alginate, the dialyzed solution was precipitated with three volumes of ice-cold ethanol and lyophilized one more time [41].

Isolation of PHB from cell biomass was carried out by extraction using chloroform for 12 h at 37 °C. The resulting extract was separated from the cell debris by filtration followed by PHB isolation from the chloroform extract via precipitation with isopropyl alcohol. The stage of dissolution in chloroform and precipitation of PHB with isopropyl alcohol was repeated at least three times. PHB was dried at 60 °C.

### 2.7. Determination of Molecular Weights of Alginate and PHB

The M*_w_* of biopolymers was determined by viscometry. Specific viscosity was calculated by the formula:

η_sp_ = (t − t_0_)/t_0_, (1)

where t_0_ is the outflow time of the solvent and t is the outflow time of the polymer solution. PHB was dissolved in chloroform, and alginate in water, respectively. M*_w_* was calculated using the Mark-Howink equation:

[η] = K(M)^a^, (2)

with coefficients for alginate:

K = 7.3 × 10^−5^; a = 0.92, [η] = 7.3 × 10^−5^ × (M)^0.92^. (3)

For PHB:

K = 7.7 × 10^−5^; a = 0.82, [η] = 7.7 × 10^−5^ × (M)^0.82^, (4)

where M is the M*_w_*, [η] is the viscosity, and K and a are constants, the value of which depends on the nature of the polymer (Alginate and PHB) [42,43].

### 2.8. FTIR Spectroscopy Characterization

IR spectra of biopolymers were recorded in reflection mode in a Hyperion-2000 IR microscope connected to an IFS-66 v/s FTIR IR spectrometer (Gecrystal, resolution 2 cm^−1^, range 4000–600 cm^−1^, scanning—50 Bruker, Billerica, MA, USA).

### 2.9. Formation of the Calcium Alginate

Calcium alginate hydrogels were produced to assess the physicochemical properties and cytotoxicity. Sodium alginate solution (1% *w/w*) was mixed with CaCl_2_ solution (10% *w/w*) at a 1:1 ratio. The volume of alginate hydrogels obtained was measured by subtracting the volume of liquid alginate and calcium chloride remaining (Vr) after polymerization from the initial total volume (Vt). The yield of the obtained polymer was calculated using the following equation [44]:

Polymer yield (%) = (Vt − Vr)/Vt × 100%.(5)

The volume of the polymer formed was dependent very closely on the total amount of guluronic acids in the alginate chain, which can bind calcium cations from solution.

### 2.10. Calcium Alginate Rheology

Experiments on the mechanical properties were carried out on an Anton Paar MCR 302 rheometer (Graz, Austria). The rheometer was equipped with a plate-plate measuring system. The typical thickness of the test samples was 3–4 mm, and the diameter was 2.5 cm.

Firstly, an amplitude test was performed to determine the range of linear viscoelasticity at an angular frequency of 10 rad/s. The characteristic range of linear viscoelasticity (0.5–1%) and the optimal gap (relative deformation 0.1–0.3) was measured, which was then followed by a frequency test being performed on another sample at an angular frequency of 0.1 to 100 rad/s. In all of the aforementioned tests, the modulus of storage G‣ and loss of G‣‣ were measured, and the complex shear modulus was calculated using the formula:

(6) $$G^{\prime \prime }=\sqrt{{\left(G^{\prime }\right)}^{2}+{\left(G^{\prime \prime }\right)}^{2}}$$

### 2.11. Water Absorption of Calcium Alginate

In assess the swelling of calcium alginates, the formed hydrogels were lyophilized for 4 h, which was then followed by the dry calcium alginate samples with constant weight (m_1_) being immersed in deionized water (25 °C) for 3 h. Wet samples were weighed after removing the water droplets. Water absorption by calcium alginate hydrogel was calculated using the formula:

A = (m_2_ − m_1_)/m_1_ × 100, (7)

where m_1_ and m_2_ are the masses of the dry and water-saturated sample, respectively.

### 2.12. Isolation and Characterization of Mesenchymal Stem Cells

All of the experiments and surgical procedures were performed in accordance with the ethical guidelines issued by the ISO 10993–1:2009 and approved by the local Bioethics Committees of Faculty of Biology, M.V. Lomonosov Moscow State University (#16.1 dated 28 May 2021). Mesenchymal stem cells (MSCs) were isolated from femurs of young (3–5 days old) Wistar rats and cultured for 2 weeks in DMEM (Dubecco’s Modified Eagle Medium, PanEco, Russia), which was supplemented with 10% fetal calf serum (FCS, Biological Industries, Israel) and 100 U/mL penicillin. MSCs were cultivated for three passages. The cells were removed via incubation in a trypsin–versene solution for 5 min and then counted using hemocytometer. Later on, a 10^5^ cell suspension in 100 μL of PE buffer (2 mL EDTA-0.5% ETS in PBS) was prepared and incubated with antibodies to positive surface markers of MSC phenotype: CD90 and CD29; negative surface markers: CD45 and CD11b/c (eBioscience, San Diego, CA, USA); the dye cell viability 7-Aminoactinomycin D (7AAD) in the dark for 40 min at a temperature of 5 °C. Cells were then washed in PBS once and extracted via centrifugation, which was followed by a flow cytometer examination (FACS ARIA II, Franklin Lakes, NJ, USA). Analysis of the results and graphs were performed using the Flowing Software 2.5.1 (Supplemental Materials, Figure S2).

### 2.13. Cytotoxicity Test

To evaluate cytotoxicity, three types of bacterial alginates were selected, namely free alginate, low molecular weight (212 kDa) capsular alginate, and high molecular weight (574 kDa) capsular alginate. For comparison with alginates of bacterial origin, alginate of algae from Sigma Aldrich with an average viscosity of 20,000–40,000 cps (about 155 kDa) was used. Cells grown on a cultural plastic (TCPS) were used as negative control (NC). For the cultivation of MSCs on TCPS in the presence of alginate hydrogel, alginate was prepared in form of spheres with a volume of 25 μL by adding a 1% alginate solution to a 10% calcium chloride solution drop wise. Subsequently, calcium alginate spheres were washed in a phosphate-buffered saline (PBS) [45]. MSCs were then incubated in a thermostat at 37 °C in a humidified atmosphere of 5% CO_2_ in a growth medium containing modified Eagle’s medium (DMEM, Invitrogen, Waltham, MA, USA) with a high glucose content (4.5 g/L), 10% fetal calf serum, antibiotic (100 IU/mL penicillin and 100 μg/mL streptomycin solution). After three passages, cells were harvested, centrifuged, and washed as mentioned in Section 2.12, which was then followed by cell seeding in a 96-well plate at 4000 cells per well. The growth medium volume per well was 200 µL. After 2 h, one calcium alginate sphere was added to each well. Cell growth on TCPS in the presence of alginate hydrogel spheres for days one and three was assessed via the XTT method using the XTT-proliferation kit (Biological Industries, Kibbutz Beit Haemek, Israel). This method is based on the ability of living cells’ active mitochondria to convert tetrazolium salts into formazan salts. To achieve this 50 µL of XTT reagent solution and 50 µL of serum-free DMEM medium were added to each well after removing the calcium alginate from the wells at the end of cultivation. The plates were incubated at 37 °C for additional 2 h. Thereafter, the absorbance of the samples was measured spectrophotometrically at 450 nm by subtracting the background absorbance at 620 nm. The number of viable cells on the films was then determined from a calibration curve based on their XTT reactivity. The results were represented as growth of viable cells in relation to the positive control, where no alginates were added to the latter.

### 2.14. Statistical Analysis

A non-parametric Kruskal–Wallis test was implemented for statistical data evaluation using the tidyverse package in the R environment (version 3.6.3, Lucent Technologies, Murray Hill, NJ, USA, codenamed “Holding the Windsock” Copyright © 2020. The R Foundation for Statistical Computing). Data obtained was presented as mean ± SD (standard error of the mean). For the analysis of obtained results of the two level three factor (2^3^), an FDD was carried out by the method of multivariate analysis of variance (three-way ANOVA) using the stats package in the R programming language. The selection criterion for significant differences between groups was *p* ≤ 0.05.

## 3. Results and Discussion

### 3.1. Description of the Producer Strain and 16S Analysis

Cultural and morphological characteristics of the strain were as follows: colonies were large, slimy, of a dark orange colour; cells were pleomorphic, mostly oblong, gram-negative, motile (with flagella), located singly, in pairs and in chains, strict aerobes (Supplemental Materials, Figure S1). Analysis of 16S ribosomal RNA confirmed that the organism from our collection belongs to the bacterial species *Azotobacter vinelandii* (https://www.ncbi.nlm.nih.gov/nuccore/MN977323.1, accessed on 25 February 2021).

### 3.2. Cultivation of Bacteria

**Figure 2:**
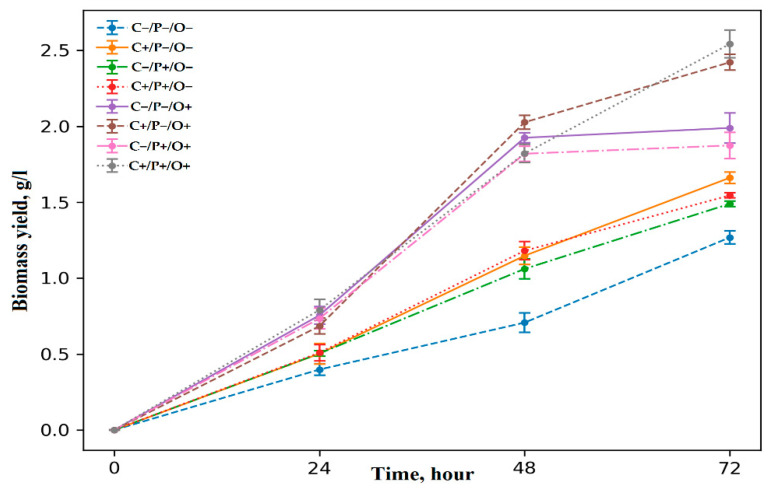
Growth dynamics of *Azotobacter vinelandii* 12 in an FFD.

The growth dynamics of the *Azotobacter vinelandii* 12 producing strain depending on variation of cultivation conditions performed by FFD is shown in Figure 2.

**Figure 3:**
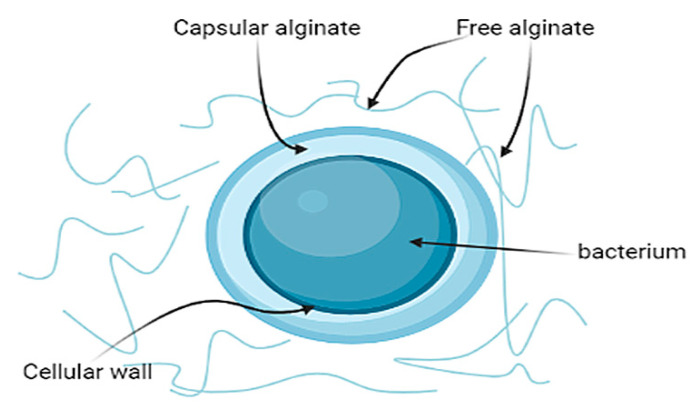
Two types of alginates synthesized by bacteria of the *Azotobacter* sp. genus: free and capsular alginates.

As can be seen from the results presented in Figure 3, there is a clear boundary that can be drawn between experiments with low aeration (O−) and high aeration (O+). These results clearly show that bacteria are more prone to division at a high level of aeration (high oxygen concentration), which is due to the fact that all bacteria of the *Azotobacter* sp. genus are strict aerobes [13]. From the significance of differences at *p* < 0.05, it can be concluded that bacteria grown in conditions with high aeration and low sucrose concentration in cultural medium do not have sufficient carbon source for constant exponential growth, and, therefore, after 48 h they enter the stationary phase (C−/O+). It can be assumed that the role of carbon as the main source for bacteria growth increases due to high agitation rate (O+) [46]. So, Barrera and his colleagues demonstrated that upon elevation of the aeration level, *A. vinelandii* begins to consume carbon as much as possible, where 25% of it is spent on the biosynthesis of alginate [47]. In many studies, it was shown that phosphates restriction in the medium significantly inhibited growth and reduced the survival of *Azotobacter* sp. [48,49]. Tsai et al. also showed that upon phosphate deficiency in growth medium, a cell wall disruption and absence of cysts becomes apparent [50]. However, the relationship between phosphate concentration and bacterial cell growth was not observed, which also includes sucrose concentration and aeration level in the experiment.

### 3.3. Synthesis of Alginates and PHB

Alginate can be divided into two types according to its functions and physicochemical properties: free and capsular alginate (Figure 3). Free alginate is synthesized into the intercellular space, has a low molecular weight, reduces oxygen concentration, and hinders diffusion within the culture medium [11]. Capsular alginate performs a protective function by covering the bacterial cell wall with a dense polysaccharide layer. This polymer has a high molecular weight and allows cell colonies to form cysts necessary for resisting adverse conditions, primarily from the effect of excess oxygen concentration on nitrogenase complexes [31].

**Figure 4:**
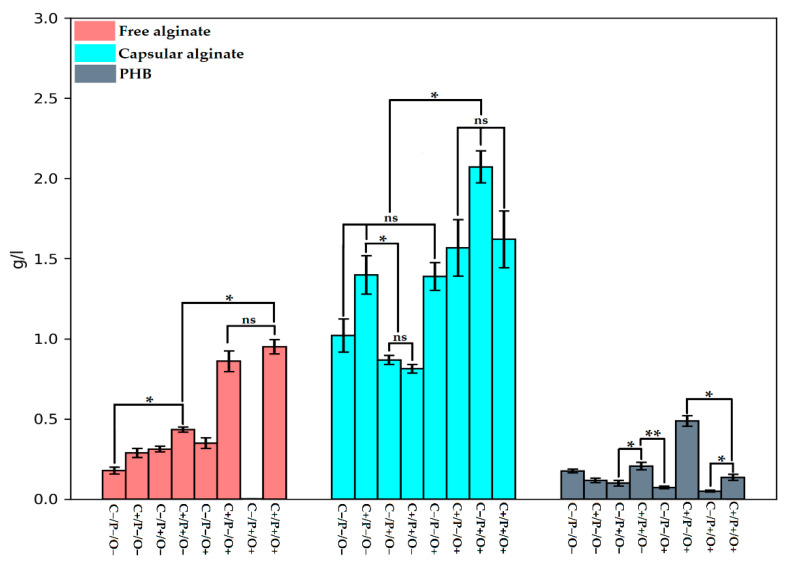
Total yield of free alginate, capsular alginate and PHB in FFD. Pairwise comparison between different groups shows the reliability level: ns, not significant; *—*p* < 0.05 and **—*p* < 0.005.

The results of bacterial biosynthesis of free alginate, capsular alginate, and PHB are shown in Figure 4. As can be seen from the graph, the maximal values of the free alginate yield are achieved at high sucrose concentrations and at a high level of aeration (C+/O+). It is also worth noting that bacteria grown in presence of sucrose deficiency, high phosphate concentration, and low aeration levels (C−/P+/O+) reached a maximum yield of capsular alginate synthesis with complete absence of free alginate synthesis. Supposably, phosphates are very important in Bourke’s medium for the formation of capsules [50], which, in turn, consist of high molecular weight alginate. This fact is of great biotechnological interest since a rare case of a possibility can be observed here, when only high molecular weight capsular alginate without admixture of low molecular weight free alginate can be obtained. The PHB synthesis was very different in all 8 variants of the FFD, but only the combination of high sugar and oxygen levels (C+/P−/O+) contributes to the effective synthesis of biopolymer—0.49 g/L (Figure 4).

As can be seen from the data presented in Figure 3 and Figure 4, all of the investigated factors largely impact the synthesis of either polymer. In our previous works, it was clearly evaluated that the *Azotobacter* sp. bacteria synthesize two types of alginates (free and capsular), which is distinguished by their physicochemical properties and functions [11,29]. In the study by Flores, it was shown that increased concentration of dissolved oxygen (DO) elevated the rate of consumption of various carbon sources by the *Azotobacter* sp. bacteria. [51]. Some FFD variants (C+/O+) showed the best growth dynamics of *A. vinelandi* 12 (Figure 2). Although the concentration of DO increased under a high level of aeration, the bacteria had enough concentration of sucrose in the medium for stable growth, and it also could use sucrose as the main carbon source for the free alginate synthesis more efficiently. Moreover, a study reported that maximum activity of alginate lyases was achieved in the pre-stationary phase [52], and, as can be seen from Figure 3, C+/P−/O+ and C+/P+/O+ are the only variants, which did not reach a plateau. In turn, it is known that at high DO concentrations the alginate lyase enzyme activity (algL, alyA1, alyA2, alyA3, and algE7) elevates and the polymerase genes expression decreases [51]. In addition, a study by Jimenez has shown that a specifically high cell growth rate promotes the low molecular weight synthesis of alginate, which was fully consistent with our data [53]. In the case of capsular alginate, it is generally known, that the alginate synthesized in the *Azotobacter* sp. bacteria accompanied by the formation of capsules is a mechanism that promotes resistance to colonies drying out of the cell in various unfavorable conditions [12,23]. For capsular alginate oxygen is the main factor since capsular alginate creates a capsule that protects the cell from toxic metal ions and oxygen, which adversely affects the activity of the nitrogenase complex, where the latter is very sensitive to molecular oxygen [31,54]. The yield of capsular alginate from the total amount of synthesized biopolymers in all different versions of the experiment ranged from 54% to 98%, where the minimum yield of capsular alginate was observed in the C+/P−/O+ variant. Thus, it can be concluded that minimum phosphorus content in the medium limited the synthesis of capsular alginate. Phosphates are an important component of the capsule formation around bacterial colonies [50], therefore, lack of phosphates prevents the capsular alginate production in optimal amounts. C−/P+/O+ version showed that almost all of the carbon source (98%) was spent on the capsular alginate synthesis with only two percent being spent on PHB. These data were also explained by the fact that phosphate actively promotes cyst formation and capsular alginate synthesis [50]. Since phosphates are the main energy source molecules for all living organisms, especially for aerobes (including *A. vinelandii* 12), a lack of phosphates might lead to unfavorable changes in cellular metabolism. Reduced phosphate concentration lowers the adenosine-5′-triphosphate (ATP) to adenosine-5′-diphosphate (ADP) ratio, which also leads to the shortened respiratory activity of bacterial cells [31], since oxidative phosphorylation is the main process of ATP synthesis (Figure 1). Presumably, a decrease in bacterial respiration can be associated with the isolated capsular alginate synthesis in the C−/P+/O+ experiment, since the protection of the nitrogenase complex proceeds through several mechanisms [31]. The first defense mechanism is the high respiratory cellular activity. In this kind of cellular respiration, oxygen is removed from the cell surface without reaching nitrogenases [55,56]. As a result, due to a decrease in respiratory activity, the main defense mechanism acts by creating dense capsules from capsular alginate, where bacterial cells lack resources for the synthesis of free alginate.

Sucrose was found to be an important factor for an improved PHB synthesis (as the main carbon source), which was consistent with the literature [57]. Oxygen level in FFD was not the key factor for an optimal PHB synthesis, since the process of PHB accumulation under controlled conditions of aeration depends on the physiological features of the *Azotobacter* sp. bacterial strain [32,58,59]. In this case, the *A. vinelandii* 12 bacterial strain is an alginate-producing strain and did not contribute to the accumulation of PHB in large quantities [11]. A small PHB amount in the C−/P+/O+ variant was associated with the presence of large amounts of capsular alginate, as the study by Reusch showed that lipid profile and the PHB amount during cyst formation reaches its peak and then falls to minimum values when cysts (capsules) around bacteria mature [60].

As a result, it was concluded that under certain cultivation conditions (C−/P+/O+), only the capsular alginate synthesis by *A. vinelandii* 12 was achieved. By only varying the cultivation parameters, the synthesis of polymers by the *A. vinelandii* 12 (alginate and PHB) culture can be regulated, as well as completely suppress the synthesis of one of them and achieve maximum yield of the other.

### 3.4. Physicochemical Properties of Alginate and PHB

The physicochemical studies data showed a large difference in M*_w_* between free and capsular alginates: free alginates in all experiments (with the exception of the C−/P+/O+ variant due to the absence of the polymer) had an M*_w_* in the region of 98–113 kDa, where, at the same time, capsular alginates’ FFD significantly differed across the experiments and also had higher M*_w_* compared to free alginate (Table 2).

**Figure 5:**
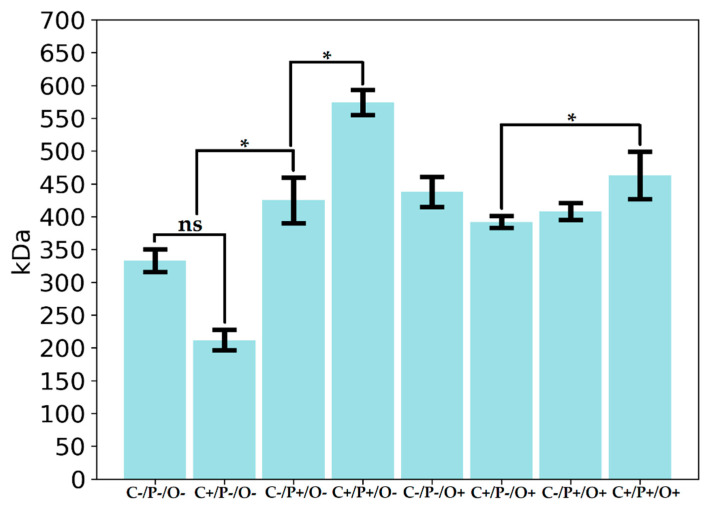
M*_w_* of capsular alginates in FFD. Pairwise comparison between different groups shows the reliability level: ns, not significant and *—*p* < 0.05.

The experiment was set up at high carbon concentration, low phosphate concentration, and low aeration level (C+/P−/O−), which resulted in capsular alginate biosynthesis with the lowest M*_w_* among all FFD experiments (212 kDa). Furthermore, the experiment involving culture growth at high concentrations of carbon and phosphates and low aeration demonstrated the highest M*_w_* of 574 kDa (Table 2, Figure 5). The effect of low aeration level on high molecular weight alginate chain polymerization was also confirmed in the study by Lozano, where the alginate synthesis with maximum M*_w_* (550 kDa) was achieved at a reduced oxygen transfer rate (OTR) [61]. The data on the correlation between the sucrose level and the capsular alginate M*_w_* were contradictory, as the experimental data of C+/P−/O− and C+/P+/O− showed minimum and maximum alginate M*_w_* at a high sucrose concentration in both cases (Figure 5).

The study of PHB M*_w_* showed diverse results (Table 2). All polymers had an M*_w_* of over 1200 kDa with the exception of C−/P−/O+: 331 kDa and C−/P+/O+: 49 kDa. It should be noted that in previous cases it was not possible to obtain such a large PHB M_*w*_ range (more than 30 times: from 49 to 1698 kDa) using our standard producing strain A. chroococcum 7B. To regulate the M*_w_* of PHB, sodium acetate was added to culture medium [3]. Therefore, this novel approach can be used to regulate the M*_w_* of PHB and its copolymers with a wider range without using any additional supplements. Apparently, a combination of high aeration and low sucrose level in the medium for the high molecular weight PHB synthesis is critical. This was also confirmed by the fact that the *A. vinelandii* 12 bacteria entered the stationary phase exactly after 48 h (Figure 3) in the C−/P−/O+ and C−/P+/O+ experiments, and, as it is known, the PHB M*_w_* varies greatly between exponential and stationary phases [62]. Thus, at the moment of exponential bacterial growth, the M*_w_* of PHB increases due to the high expression of the PhbC gene, which translates PHB synthase. Also, at the moment of bacterial cells entering the stationary phase, the depolymerase (PhbZ) gene is actively expressed, which results in the production of low molecular weight PHB (Figure 1) [62].

**Figure 6:**
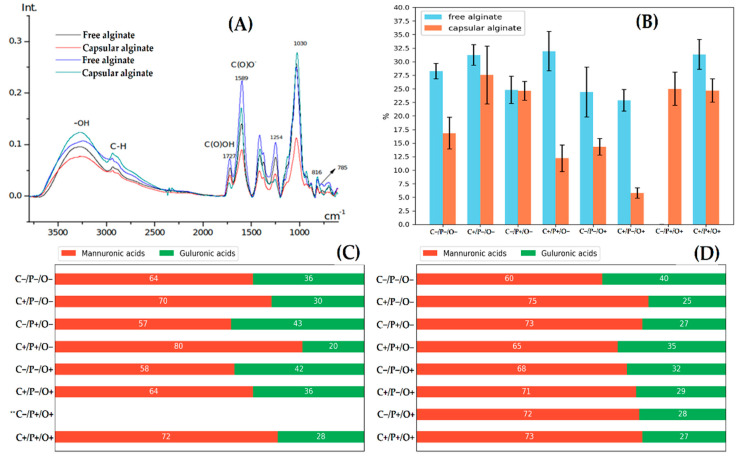
Physicochemical properties of bacterial polymers. (**A**) Absorption spectra of bacterial alginates. (**B**) Acetylation level of samples in FFD of bacterial alginates. Resides distribution of uronic acid in free (**C**) and capsular (**D**) alginate. ** Experience C−/P+/O+ of free alginate is absent due to the lack of its synthesis.

According to the IR spectra of bacterial alginates, the monomeric composition of the polymer by M/G and the acetylation level can be determined. In Figure 6A, the alginate spectra had absorption bands with different intensities at 1600 cm*^−^*^1^ (COO^−^), 1720 cm*^−^*^1^ (COCH_3_), 1030 cm*^−^*^1^ (CO), 1254 cm*^−^*^1^ (COOR), 816 cm*^−^*^1^ (M-blocks), and 785 cm*^−^*^1^ (G-blocks) [13]. Absorption bands corresponding to proteins, cell walls, or nucleic acids were not observed, which implies high purity of the obtained polymers.

The mannuronic to guluronic acid ratio in the alginate chain was determined by the 816 cm^−1^ band ratio, which shows a stretching vibration of the mannurons (M) pyranose ring to the absorption band in the 785 cm^−1^ region, which corresponded to guluronic acid (G) [63]. The alginate chain M/G percentages in all runs of free alginates and capsular alginates are shown in Figure 6C,D, respectively.

The distribution of uronic acids in free and capsular alginates showed that mannuron blocks in the polymer chain predominated in all samples. To date, there is a limited number of research investigating the effect of various factors of bacterial culture on the synthetized alginate M/G distribution. Most of the studies in this area are devoted to the functions of C5 epimerases [64,65]. There are seven enzymes (AlgE1–AlgE7) known to be responsible for epimerase activity in the *A. vinelandii* bacteria. Among all of these epimerases, only AlgE1 has a pronounced effect on creating long poly-G blocks from poly-M blocks; this activity is very useful in creating strong and durable alginate hydrogels [64]. To assess the influence of environmental factors on the distribution of synthesized alginates’ uronic acids, additional transcriptome studies on products of the C5 epimerase genes of the *A. vinelandii* 12 bacterium are required.

The acetylation level was determined by the 1600 cm^−1^ to 1720 cm^−1^ band ratio. It is known that 1030 cm^−1^ and 1254 cm^−1^ bands can also show signals of the acetyl group absorption bands [12]. The acetylation level results of free and capsular alginates are shown in Figure 6B, respectively. From the collected data, it can be noted that the acetyl group number on the mannuron residues of free alginates in all experimental variants differed slightly (23–32%) and was found to be higher in contrast to capsular alginate (6–27%).

According to the data on capsular alginates, there was a significant scatter in different experimental variants (from 6% in C+/P−/O+ experiments to 27% in C+/P−/O− experiments) in acylation level. It should be noted that C+/P−/O+ variant showed the lowest acetylation percentage (Figure 6B) with maximum PHB yield (Figure 4), and vice versa, when there was a high acetylation level (25%) a complete absence of PHB synthesis (0.027 g/g, M*_w_* = 49 kDa) in C−/P+/O+ variant was observed. Previously, Castillo et al. showed that acetyl-CoA is an acetyl donor for the alginate acetylation and also a precursor of PHB [66]. Barrera et al. came to a similar conclusion when they observed that acetyl-CoA becomes more available for alginate acetylation than for PHB synthesis under high aeration [67]. In the case of this study, in addition to aeration, sucrose and phosphates factors were also involved, where just between the C+/P−/O+ and C−/P+/O+ experiments, several differences in the sucrose concentration were observed. As previously discussed, sucrose level in the growth medium is an important indicator of PHB synthesis (Figure 1) [23]. Thus, it can be concluded that a sucrose concentration decline and an increase in the aeration level directly switch the streams of acetyl-CoA from the synthesis of PHB to the cycle of tricarboxylic acids (TCA) [27], in which acetyl-CoA is the main donor for the reproduction of acetyl groups on mannuron residues.

It is important to note that C−/P+/O+ variant, in which only capsular alginate was observed, had a high acetylation level (25%). It was assumed that under these conditions *A. vinelandii* 12 can potentially synthesize high molecular weight alginate with M/G = 70/30 and demonstrate a high acetylation level without PHB and free alginate production, which is essential for many biomedical tasks.

### 3.5. Three-Way ANOVA of FFD

**Figure 7:**
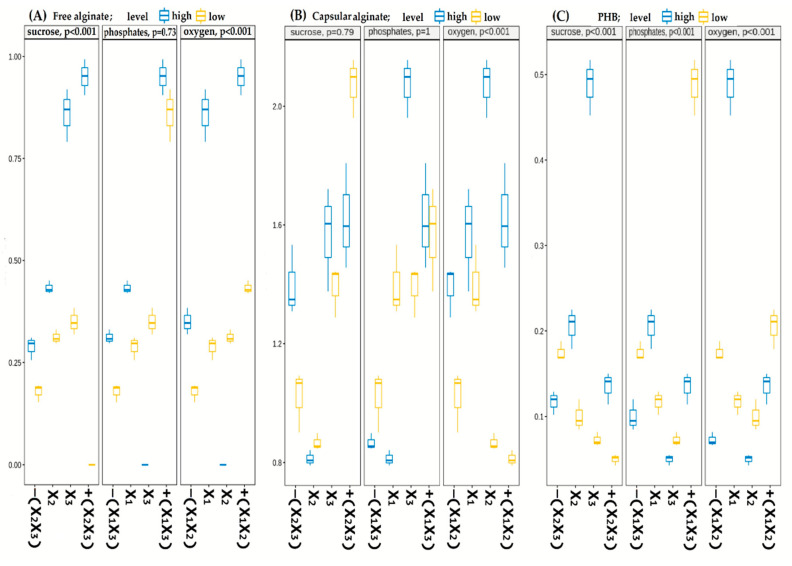
Full Factorial design (FFD 2^3^) for the synthesis of free alginate (**A**), capsular alginate (**B**), and PHB (**C**).

In this study, the significance of three factors in FFD and their interactions were screened using the three-way ANOVA method for three independent variables, such as total synthesis of free and capsular alginates as well as PHB synthesis (Figure 7). The *p*-value of less than 0.05 indicated that the model with these factors was found to be significant at the 95% confidence level [68].

Figure 7 shows that the greatest impact on biopolymer synthesis was made by the agitation rate (oxygen level) and, in the order of orthogonal variables to the total polymer synthesis according to F and *p*-values were X_3_ (oxygen) > X_1_ (sucrose) > X_2_ (phosphates). It is worth noting that only oxygen factor (X_3_) was significant (*p* < 0.05) for the synthesis of all of the polymers, whereas sucrose factor (X_1_) was found to be not significant for the capsular alginate synthesis. Furthermore, no significant differences were observed in the free and capsular alginate syntheses at various phosphate concentrations (X_2_). This can be explained by the fact that at different aeration levels the amount of dissolved oxygen in the environment will also change, which in turn alters the cellular respiration activity of the *A. vinelandii* bacteria [69]. These kinds of modifications are accompanied by a change in the activity of the nitrogenase complex in bacteria. They activate various nitrogenase protection systems from oxidation by oxygen, where one of the protectors is alginate [31].

Interaction of the FFD 2^3^ factors were also determined by the ANOVA method, which is provided in Table S1 in the Supplementary Materials section. The interactions of factors for the synthesis of free alginate showed a strong influence in all of the interactions and were arranged in the following order: X_1_X_3_ > X_2_X_3_ > X_1_X_2_ > X_1_X_2_X_3_. It is worth noting a particularly strong interaction of sucrose and oxygen factors (F-value = 502.09). Previously, according to the polymer biosynthesis data, it was already observed that positive values of carbon concentrations and aeration level of (C+/O+) induce the free alginate production to maximum values. Thus, as shown in the study by Flores et al., the maximal synthesis of the free low molecular weight alginate occurs under conditions of increased DO concentration due to high aeration (X_3_), which in turn increases the consumption rate of the carbon source by the bacteria in cultural medium (X_1_) [29].

Interactions in the capsular alginate synthesis showed that the influence of all three factors (X_1_X_2_X_3_) was not statistically significant (*p* = 0.3). Interestingly, the isolated effect of phosphates was not significant for the capsular alginate production, but its interactions with oxygen (X_2_X_3_) and sucrose (X_1_X_2_) showed the strongest impact on polymer yield. Phosphates play an important role in capsular alginate production since cyst formation did not occur without the participation of the component [50]. However, cyst formation is an important step of the life cycle of the *Azotobacter* sp. bacteria [70]. Therefore, the effect of phosphate presence on the high molecular weight capsular alginate production showed a solid response only under specific bacterial cultivation conditions, such as a high aeration level, where capsules are formed to protect nitrogenases.

The results of variance analysis on the PHB synthesis showed that all combinations of the factor influences made a significant contribution to the system (*p* < 0.05). The strongest system response to the PHB synthesis showed a simultaneous interaction of all three factors (X_1_X_2_X_3_, F-value = 265.82). These data show that the biopolymer accumulation process performed by cells is regulated by a large number of signaling molecules and regulatory proteins, which in turn interact with a cluster of synthetic PHB genes [71]. Each of the three factors (sucrose, phosphate, and oxygen) can induce the gene expression that can act both as an inhibitor and activator for the pha biosynthetic locus at the transcriptional (transcription factors) or post-transcriptional (sRNAs) level [72,73].

### 3.6. Rheological Properties of Calcium Alginate

In many mechanical properties’ studies of various hydrogels, it was shown that high molecular weight alginates and a high guluronic residue ratio in the chain create denser hydrogels [74]. In this case, after performing the synthesis using the FFD protocol, capsular alginates were found to have a high M*_w_* (M*_w_* = 574 kDa for the experiment C+/P+/O−). Free alginates in all eight experiments had an average M*_w_* in the range from 98 to 113 kDa; gelation of alginates with such a low M*_w_* is generally accompanied by limitations in the production of high-density hydrogels based on it. Therefore, rheological testing of free alginates has not been carried out.

To study the rheological properties of capsular alginates, alginates from the C+/P−/O− experiment with the lowest M*_w_* and alginate from the C+/P+/O− option with the highest M*_w_* were selected based on the data from viscosimetry and IR spectroscopy. Commercial algal alginate with an average viscosity of 20,000–40,000 cps or 155 kDa (Sigma-Aldrich, Darmstadt, Germany) was utilized as a control.

**Figure 8:**
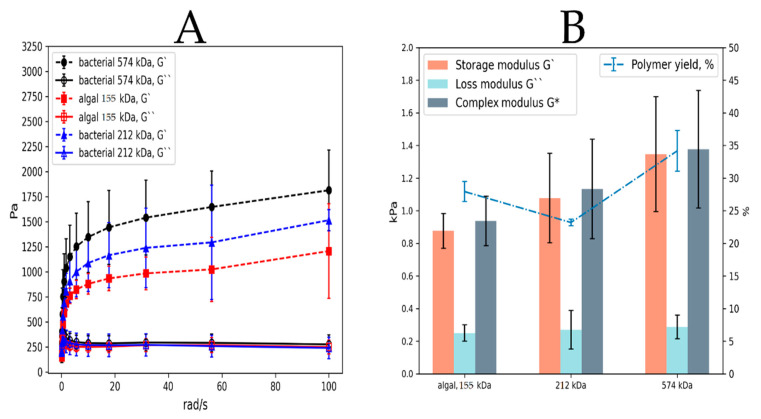
(**A**) Alginate sample frequency sweep measurements. (**B**) Correlation of the obtained viscoelasticity results at 10 rad/s with a total alginate yield during gelation.

Based on the dynamic viscoelasticity data in Figure 8, it was found that the high molecular weight capsular alginate (574 kDa) produced hydrogels with the highest density. The storage modulus (G‣) shows the applied stress on the gel (in phase with the strain) to the magnitude of that strain ratio. This parameter directly correlates with the ability of alginate to form ionotropic networks of guluronic residues with calcium, thereby showing the amount of Ca-Gul crosslinks [75].

Figure 8A demonstrates a frequency variation graph of the alginate gels with different physicochemical properties and different polymer origins (algal and bacterial). Although high molecular weight capsular bacterial alginate produces the densest gels, there was no linear correlation with algal and low molecular weight capsular alginate. A non-linear relationship between gel strength and calcium addition arose as a result of the crosslinking mode between the alginate chains [76]. At low counterion addition levels, there is a limited replacement of sodium with calcium ions, which leads to a low crosslink density [77]. In addition, the random nature of the guluronic monomer arrangement in the chain disturbs the creation of dense homogeneous gels due to the formation of tightly cross-linked and aggregated regions inside the hydrogel [78]. Such poor network development results in aggregated particles with an overall reduced network strength [76]. With a frequency decline of all three polymers to 0.1 rad/s, it can be seen that the loss modulus (G‣‣) (that shows the energy part that goes into heat in one oscillation period) becomes greater than G‣ (G‣ < G‣‣). In many studies on viscoelastic properties of alginate it has been shown that both modules begin to intersect when low frequencies of 0.5 rad/s and below are reached [79,80]. Such data indicate that the hydrogels obtained had more rheological properties of a liquid than of a solid, which corresponded to literature data [80].

The rheological data results at constant frequency (10 rad/s) and polymer yield did not have a direct relationship (Figure 8B and Table 3). Although the average values of the complex modulus (G*) (which is calculated from G‣ and G‣‣) were different for all polymers, it cannot be fully confirmed that there were any differences between the three samples according to one-way ANOVA (*p* > 0.05). However, the polymer yield in presence of alginate gelation was different between all three samples (Figure 8B and Table 3).

Due to its high M*_w_* (574 kDa), capsular alginate demonstrated the highest polymer yield upon mixing alginate and calcium chloride solutions. Although the resulting relation was non-linear, there was a direct correlation between the calcium alginate hydrogel formation and the M*_w_* (r = 0.83). Chaotic ionotropic binding of guluronic residues in polymers showed a non-linear relationship to the M*_w_* of alginate. Low M*_w_* (212 kDa) capsular alginate had the lowest polymer yield (23.19%) primarily due to a small amount of guluronic monomers in the chain (M/G = 75/25). Therefore, it can be assumed that M*_w_* and M/G content are the limiting parameters in the creation of hydrogels.

Several differences in water absorption and swelling ratio were found between the 574 kDa capsular alginate and the other two polymers (algal alginate and 212 kDa capsular alginate) (Table 3). A higher degree of capsular alginate swelling from the fourth variant of FFD was achieved due to its high molecular weight. Also, as a result of the higher M*_w_* alginate creates more gulurone crosslinking points with calcium ions in the primary alginate chain, thereby creating a more extensive polymer network for penetration of water molecules [75]. Moreover, long alginate polysaccharide chains are proportional to their viscosity, and high viscosity alginates can form stronger hydrogels, as polysaccharide chains are more likely to crosslink and physically entangle [81].

### 3.7. Calcium Alginate Cytotoxicity Test

**Figure 9:**
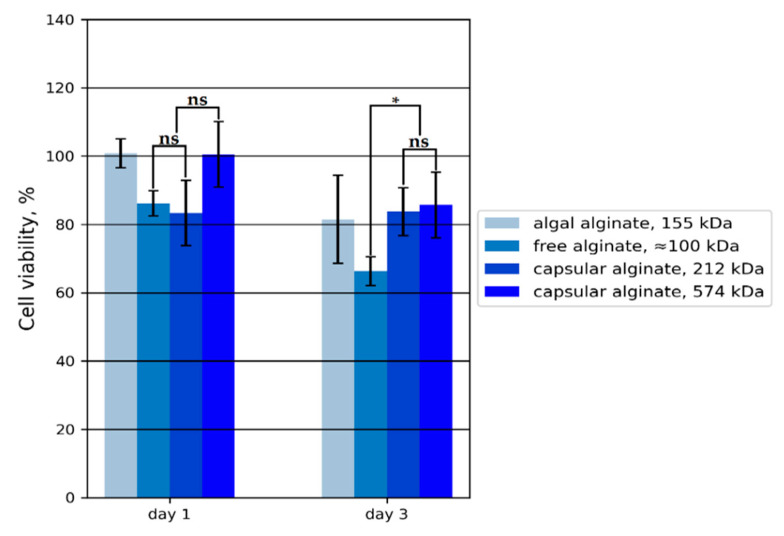
The proliferation rate of MSCs. Cell viability as a percentage was calculated relative to the negative control (NC). Pairwise comparison between different groups shows the reliability level: ns, not significant and *—*p* < 0.05.

Calcium alginate hydrogels have been tested for cytotoxicity with regards to future use of the bacterial polymer for in vivo applications in tissue engineering (Figure 9).

The algal and high molecular weight capsular alginate hydrogel data did not demonstrate cytotoxicity in mesenchymal stem cells (MSCs) and were comparable to the first-day control. Free and low molecular weight capsular alginate-based hydrogels showed a trend (statistically insignificant), where negligible toxicity (over 80% of NC) for MSCs was observed. On the third day of cell growth, all alginates exhibited insignificant toxicity to MSCs, except for free alginate, where the number of viable cells was found to be less than 70%. A possibly low alginate Mw (as in free alginate) does not promote active cell growth. At the moment, there is little information on the effect of Mw of alginates on cytotoxicity and biocompatibility. Based on these data, it can be assumed that alginates with the lowest Mw have the most striking toxic effect on cells.

## 4. Conclusions

In the current study, a general assessment of the effect of sucrose, phosphates, and oxygen on the synthesis and physicochemical properties of bacterial PHB and alginates in PFD was conducted. The effect of oxygen factor (based on aeration level) on the polymer synthesis had a greater response in contrast to factors of sucrose and phosphates. By controlling the conditions, it is possible to achieve the synthesis of either polymer with properties suitable for specific biomedical tasks. Therefore, under a reduced sucrose concentration and an increased phosphate concentration in the culture medium, as well as a high aeration level, an isolated synthesis of high molecular weight capsular alginate can be achieved. High molecular weight capsular alginate has better rheological properties and is less cytotoxic to MSCs compared to low molecular weight free alginate and can be easily compared to commercial algal alginate. Further studies of bacterial alginates will be useful in the tissue engineering field since the use of alginate hydrogel structures for the regeneration of various tissues and organs is in high demand nowadays [82,83]. Also, as known from numerous studies, alginates of bacterial origin demonstrate better alginates mechanical properties and biocompatibility in comparison to algae [41,84], which also includes its controlled synthesis [11], therefore making it possible to improve the technologies of hydrogel and scaffold development in tissue engineering.

## Figures and Tables

**Table 1:** Full factorial design (FFD).

	C−/ P−/O−	C+/ P−/O−	C−/ P+/O−	C+/ P+/O−	C−/ P−/O+	C+/ P−/O+	C−/ P+/O+	C+/ P+/O+
Sucrose g/L (X_1_) (+) 35 (−) 15	−	+	−	+	−	+	−	+
Phosphates g/L (X_2_) (+) 1.25 (−) 0.05	−	−	+	+	−	−	+	+
Agitation rate (O_2_), rpm (X_3_) (+) 210 (−) 150	−	−	−	−	+	+	+	+

**Table 2:** Molecular weights (M_w_) of free alginate, capsular alginates, and PHB.

FFD	Free Alginate, kDa	Capsular Alginate, kDa	PHB, kDa
C−/P−/O−	113 ± 3	333 ± 17	1692 ± 28
C+/P−/O−	103 ± 3	212 ± 15	1296 ± 15
C−/P+/O−	106 ± 5	425 ± 34	1225 ± 9
C+/P+/O−	100 ± 2	574 ± 19	1698 ± 33
C−/P−/O+	98 ± 4	438 ± 23	331 ± 9
C+/P−/O+	110 ± 3	392 ± 9	1645 ± 30
C−/P+/O+	0	408 ± 12	49 ± 2
C+/P+/O+	104 ± 5	463 ± 36	1374 ± 21

**Table 3:** Alginate mechanical properties. Pairwise comparison between different groups shows the reliability level: ns, not significant and *—*p* < 0.05.

Alginates	Polymer Yield, %	Water Absorption, %	G* at 10 rad/s, kPa
Algal 155 kDa	27.92 ± 1.55	232.57 ± 13.32	0.94 ± 0.15
Bacterial 212 kDa	23.19 ± 0.52	231.50 ± 22.72	1.13 ± 0.31
Bacterial 574 kDa	34.17 ± 3.12	341.45 ± 21.89	1.38 ± 0.36
Algal 155 kDa: Bacterial 212 kDa	ns	ns	ns
Algal 155 kDa: Bacterial 574 kDa	ns	*	ns
Bacterial 212 kDa: Bacterial 574 kDa	*	*	ns

## Data Availability

Data sharing not applicable.

## Supplementary Materials

The following are available online at https://www.mdpi.com/article/10.3390/polym14010131/s1. Figure S1. Cell colonies of *Azotobacter vinelandii* 12; Figure S2. Data of cytometry of MSCs isolated from rat bone marrow; Table S1. Three-way ANOVA statistics for the yield of bacterial biopolymers (free alginate, capsular alginate and PHB). The significance of individual factors and their interactions are presented in the form of asterisks: * —*p* < 0.05, **—*p* < 0.01, ***—*p* < 0.001.
