# Supplementary material for: Competitive Biosynthesis of Bacterial Alginate Using Azotobacter vinelandii 12 for Tissue Engineering Applications
Source: Polymers (Basel). 2021 Dec 30;14(1):131. doi: 10.3390/polym14010131 (PMC8747204; doi:10.3390/polym14010131)
Supplement: Supplementary file 1 [file polymers-14-00131-s001.zip › polymers-1483450-supplementary.pdf]

Supplementary Materials:

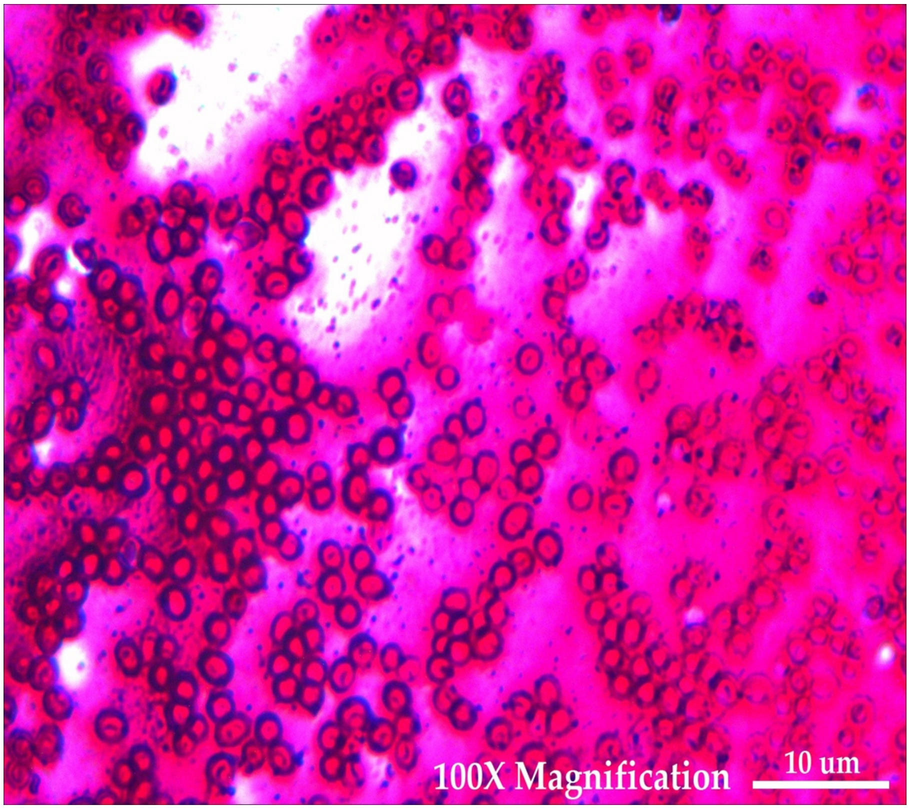

Figure S1. Cell colonies of *Azotobacter vinelandii* 12.

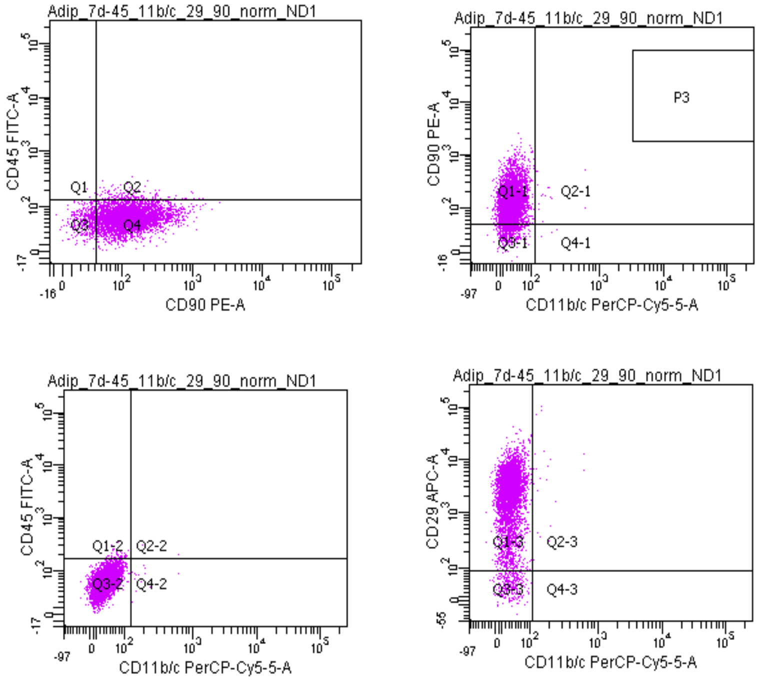

Figure S2. Data of cytometry of MSCs isolated from rat bone marrow.

Table S1. A three-way ANOVA statistic for the yield of bacterial biopolymers (free alginate, capsular alginate and PHB). The significance of individual factors and their interactions are presented in the form of asterisks: not significant – NC, * —p <0.05, ** - p <0.01, *** - p <0.001.

		**Free alginate**		
**Factors**	**Mean Sq**	**F value**	**Pr(>F)**	**Strength**
Sucrose (X_1)	1.07	950.52	1.12e-15	***
Phosphates (X_2)	0.0001	0.12	0.73	NC
Oxygen (X_3)	0.34	299.37	8.82e-12	***
X_1X_2	0.08	67.61	3.88e-07	***
X_1X_3	0.57	502.09	1.65e-13	***
X_2X_3	0.11	96.57	3.50e-08	***
$X_1X_2X_3$	0.0690	61.18	7.42e-07	***
		**Capsular alginate**		
**Factors**	**Mean Sq**	**F value**	**Pr(>F)**	**Strength**
Sucrose (X_1)	0.0009	0.07	0.79	NC
Phosphates (X_2)	0.00	0.00	1.00	NC
Oxygen (X_3)	2.44	183.52	3.49e-10	***
X_1X_2	0.42	31.83	3.68e-05	***
X_1X_3	0.13	10.02	0.00599	**
X_2X_3	0.81	61.32	7.31e-07	***
$X_1X_2X_3$	0.01	1.13	0.30	NC
		**PHB**		
**Factors**	**Mean Sq**	**F value**	**Pr(>F)**	**Strength**
Sucrose (X_1)	0.11	327.24	4.47e-12	***
Phosphates (X_2)	0.04	144.12	2.05e-09	***
Oxygen (X_3)	0.01	24.28	0.000151	***

X_1X_2	0.01	30.50	4.63e-05	***
X_1X_3	0.07	224.44	7.79e-11	***
X_2X_3	0.06	165.50	7.46e-10	***
$X_1X_2X_3$	0.09	265.82	2.18e-11	***
